# A Case of Aneurism of the Orbit

**Published:** 1870-05

**Authors:** E. Powell

**Affiliations:** Prof. of Military Surgery and Surgical Anatomy, in Rush Medical College


ARTICLE III. — *A Case of Aneurism of the Orbit.* — By E. POWELL, M. D., Prof. of Military Surgery and Surgical Anatomy, in Rush Medical College.

N. J. K., 12 years of age, from the State of Iowa, consulted Dr. E. L. Holmes, Oct. 29, 1869, at the Chicago Charitable Eye and Ear Infirmary, for a peculiar pulsating tumor of the left orbit.

After making a careful examination of the case, Dr. Holmes referred the patient to my care, with the following history: The patient had always been strong and in good health, and yet not quite so robust as his six brothers and sisters. There was no apparent cause of the disease, either accidental or hereditary. On the evening of August 30, 1869, he was seized with nausea and vomiting, which continued through the night and the following day. This attack could only be ascribed to the efforts required to lift a pail of water quite high, just before experiencing the nausea. About two o'clock, on the morning of September 1st, the patient was seized with an agonizing pain over the left eye. The next morning the parents observed a very great degree of exophthalmos. The globe was immovable and the cornea almost entirely uncovered. The patient suffered constant and intense pain over the eye, with fever, nausea and delirium, during three weeks. At the end of this time the lower lid began to be everted, its conjunctival membrane becoming enormously swollen and red; but the fever and nausea gradually subsided, the latter recurring for a short period every two days. The pain had been controled by the use of quite large doses of morphine. When the patient came to the Infirmary he was pale, emaciated and weak, without appetite, but with no pain, and with only a slight tendency to nausea. The pulse was 110 and very feeble. There was very great exophthalmos, with scarcely any motion of the upper lid or globe. The cornea was partially covered by the lid, and was less sensitive to the touch than normal. The lower lid was completely everted, its conjunctiva being very œdematous and red. The ocular conjunctiva was much congested but not œdematous.

The sound peculiar to aneurism was heard by the ear placed on any part of the head. The characteristic pulsation and thrill were also perceived on placing the finger on the globe, but especially just under the nasal portion of the brow. Compression of the left common carotid arrested this peculiar sound and pulsation.

The central portion of the left cheek was much swollen. On placing the finger in the mouth and grasping the cheek between it and the thumb, the same pulsation and thrill were perceived along the course of the enlarged facial artery.

The pupil was dilated and immovable; vision being reduced to about one-third of its normal distinctness, the field of vision was normal in extent. The ophthalmoscope revealed no pulsation or appearances differing in the least from those of the opposite eye, in which vision was perfect.

In counsel with Profs. J. W. Freer and H. A. Johnson, the consulting surgeons of the Infirmary, it was decided that the ligation of the common carotid was indicated. The father of the patient, however, would not consent to this operation, and took his son home; but returned with him for an operation January 10th. The pain and nausea had wholly subsided; the appetite, strength and general appearance were excellent. Pulse 75, and strong. The local symptoms remained unchanged, except that the exophthalmos was perhaps slightly increased, and the lower part of the cornea had become somewhat opaque. The cornea was less sensitive to the touch than before. Dr. Holmes stated that no abnormal, intraocular appearances could be detected with the ophthalmoscope.

I determined at once to tie the common carotid, and subsequently, in case the aneurism should reappear, by means of hot irons and injections of a solution of lactate of iron, overcome the tumor, as in a similar case, treated successfully, and reported in the London *Lancet*, vol. 2, 1853, in which I had assisted the late Prof. D. Brainard.

The common carotid was ligated January 13th, just below the crossing of the Omo-hyoid. The patient being under the influence of ether, scarcely any blood was lost. The pulsation and thrill in the orbit and cheek ceased immediately. For the first two days the patient's condition seemed most favorable. On the morning of the 15th his mother found that the right arm and leg were totally paralyzed. It is impossible to state precisely when the paralysis occurred, although there were evidences of mental aberration the day before; an examination of his limbs was neglected. On the same day distinct rales were observed throughout the whole chest.

At eleven o'clock, on the morning of the 18th, there was an alarming hemorrhage from the wound in the neck. The patient being placed at once under the influence of ether, search was made for the origin of the hemorrhage. It could not, however, be discovered, although the wound was carefully opened. As no more blood escaped, the wound was re-closed. No further hemorrhage occurred. On the evening of the 19th, the pulsation and thrill re-appeared in the orbit, at first feebly, and then nearly as strong as in the beginning. There had always been a regular discharge of urine. Evacuations of the bowels were obtained by enemata. Subsequent to the operation the patient had been bountifully supplied with the most nutritious forms of liquid diet. The symptoms of coma, the difficulty of breathing, and the frequency and feebleness of the pulse constantly increased to the day of his death, which occurred January 21st. No autopsy was permitted.

